# Effects of Alanyl-Glutamine Dipeptide Supplementation on Growth Performance, Nutrient Digestibility, Digestive Enzyme Activity, Immunity, and Antioxidant Status in Growing Laying Hens

**DOI:** 10.3390/ani14202934

**Published:** 2024-10-11

**Authors:** Usman Nazir, Zhenming Fu, Xucheng Zheng, Muhammad Hammad Zafar, Yuanjing Chen, Zhi Yang, Zhiyue Wang, Haiming Yang

**Affiliations:** 1 College of Animal Science and Technology, Yangzhou University, Yangzhou 225009, China; 2 College of Veterinary Medicine, Yangzhou University, Yangzhou 225009, China; 3 Joint International Research Laboratory of Agriculture and Agri-Product Safety of Ministry of Education of China, Yangzhou University, Yangzhou 225009, China

**Keywords:** alanyl-glutamine, growth, immunity, antioxidant

## Abstract

**Simple Summary:**

Alanyl-glutamine dipeptide carries utmost importance for the poultry industry due to its potential for enhancing various aspects of health and performance in laying birds. Glutamine is an important energy source for highly prolific immune cells and intestinal epithelial cells, and it plays a role in protein degradation, cell defense, cell repair, and is a precursor of glutathione. By improving growth, nutrient digestibility, digestive enzyme activity, immunity and antioxidant status, it can lead to the development of more productive and stress tolerant laying birds which can ultimately benefit poultry producers and consumers by reducing feed costs, mortality rates and improving egg production.

**Abstract:**

Alanyl-glutamine (**Aln-Gln**), a highly soluble and stable Glutamine-dipeptide, is known to improve the performance of poultry birds. This study aimed to investigate the effect of Aln-Gln during the rearing period on growth performance, nutrient digestibility, digestive enzyme activity, immunity, antioxidant status and relative gene expression of *Hy-Line* brown hens. A total of 480 healthy day-old *Hy-line* brown chicks with similar body weights were randomly divided into four dietary groups (8 replicates/group and 15 birds/replicate). Groups A, B, C and D were fed diets containing 0%, 0.1%, 0.2% and 0.3% Aln-Gln, respectively, for 6 weeks. The body weight (**BW**) and average daily gain (**ADG**) were higher in hens fed test diets compared with the control (*p* < 0.05). The feed conversion ratio (**FCR**) was better in test groups as compared to the control group (*p* < 0.05). The ADFI showed no significant difference between the groups. Dietary treatments had no effect on dry matter (**DM**), organic matter (**OM**) and crude fiber (**CF**) digestibility. The Aln-Gln also improved gross energy (**GE**) and crude protein (**CP**) digestibility (*p* < 0.05). It has also increased IgG levels in groups C and D. IgM levels were similar to the control in B, C and D. The Aln-Gln increased IL-1 in B and C, IL-2 in C and D, and IL-6 in all test groups (*p* < 0.05). The supplementation of Aln-Gln had no effect on serum antioxidant indices like CAT, MDA, GSH-PX, GSH, and SOD in 42-day-old growing hens. Aln-Gln supplementation had no significant effect (*p* > 0.05) on the activity of amylase and lipase, however, a significant improvement (*p* < 0.05) in the activities of trypsin and chymotrypsin was observed in the test groups. Supplemented Aln-Gln levels in the birds’ diets led to an increase in the expression of genes related to growth factors (IGF-1, IGFBP-5), immune markers (IL-1, IL-2, IL-6) and antioxidant status (GSH-Px1), as compared to control group. Aln-Gln supplementation in *Hy-Line* brown hens during their growing period improved growth, nutrient digestibility, immunity and digestive enzymes activity. These findings suggest that Aln-Gln is a promising dietary additive for enhancing poultry performance.

## 1. Introduction

Poultry growth, production, enzymatic activity and immunity mainly rely on proteins. The building blocks of these proteins are amino acids (**AA**), which are also fundamental components of eggs and meat [1]. To maximize feed efficiency in poultry production, it is essential to maintain a balanced and appropriate intake of AA [2]. During the growing period, the health and reproductive development of hens are vital factors that have a direct influence on their production performance during the laying period [3]. The size, weight, and overall health of growing hens directly impact egg production and livability, directly affecting the cost-effectiveness of poultry businesses. Providing proper nutrition and care during the growing period is essential to ensure higher profitability in poultry farming [4].

Glutamine (Gln) is a precursor for synthesizing arginine and proline, the constituents of body proteins, and is considered a key AA in the feed of growing hens. It upregulates the expression of genes necessary for cell growth and downregulates those related to immunity and oxidative stress [5]. The Gln reserves within birds’ bodies are not enough for coping with disease and stressed conditions and it is necessary to supplement it in the feed. The utilization of free Gln in diets is not efficient [6]. Alanyl-glutamine dipeptide is suggested to be an alternative to Gln monomers due to its increased stability and solubility compared to free Gln [7].

An animal’s nutritional status exerts a significant impact on antioxidant capacity, immune responses and programmed cell death. Various functional nutrients participate in the development and functional activation of immune cells either by directly regulating the response of white blood cells against infectious activities or indirectly affecting the endocrine system during microbial invasion [8]. Antioxidant nutrients play a vital role as a protective shield against reactive oxygen species by inhibiting lipid peroxidation and oxidative chain reactions and enhancing the production of antioxidant enzymes [9]. Deficiency of a specific nutrient can also lead to regulation of autophagy and cell apoptosis. Amino acids, building blocks of proteins, can also upregulate various pathways through direct signaling, which leads them to play diverse roles within the body [10]. Glutamine is found in abundant quantities in the blood and is important for maintaining gut integrity by supporting protein synthesis, regulating immune and antioxidant response, promoting cell proliferation and delaying cell death [11]. Furthermore, it also activates several types of kinases related to cell signaling. Moreover, it also increases intestinal cell proliferation through MAPK activation, while the upregulation of the mTOR pathway improves protein synthesis and also improves cell survival through the regulation of heat shock proteins in the intestine [12,13]. Glutamine carries utmost importance as a major energy substrate for the development of enterocytes. Almost 70% of the Gln can be degraded within the small intestines of rats and pigs during the first pass. However, its deficiency may induce autophagy, which affects mTOR and MAPK signaling pathways in porcine intestinal epithelial cells [14,15,16].

Based on the findings of this study, the hypothesis tested was that supplementing Hy-line Brown hens with Aln-Gln during the rearing period will lead to sustained improvements in growth performance, nutrient digestibility, digestive enzyme activity, immunity, and antioxidant status, ultimately resulting in improved birds’ health and a finding that could significantly impact the poultry industry.

## 2. Materials and Methods

### 2.1. Experimental Design, Birds and Diets

A total of 480, one-day-old *Hy-line* Brown chicks were assigned equally to 4 groups with 8 replicates of 15 chicks each. All chicks were kept in cages (37 × 30 × 40 cm) that were equipped with external access devices for water and feed throughout the trail and raised according to the Management Guide of *Hy-line* brown hens [17]. The test diets were formulated using the soya–corn basal diets supplemented with 0% (control), 0.1%, 0.2% and 0.3% Aln-Gln (groups A, B, C and D). The diets were formulated as shown (Table 1) to meet the nutritional requirements of chicks according to recommendations of [18].

A powder form of Aln-Gln (Shandong Chen-long Pharmaceutical Co., Ltd., Jining, China) was added to the diets.

### 2.2. Growth Performance 

The feed intake and body weight of all the replicates were recorded on a weekly basis, while the phase-wise calculation of body weight (BW), the average daily gain (ADG), average daily feed intake (ADFI) and feed conversion ratio (FCR) was performed at 0–2 weeks, 3–4 weeks, 5–6 weeks and 0–6 weeks. Phase selection was used to observe the impact of rearing periods on growth performance. Moreover, the above parameters were also corrected for mortality.

### 2.3. Digestive Enzyme Activity Assay

Following the feeding trial, one bird was randomly selected from each replicate (8 birds per group) for the purpose of slaughter. Specimens of duodenal chyme were collected and preserved at a temperature of −80 °C. The levels of amylase (Kit cat. No. C016-1-1), lipase (Kit cat. No. A054-2-1), chymotrypsin (Kit cat. No. A080-3-1), and trypsin (Kit cat. No. A080-2-2) in duodenal samples were measured using commercially available kits provided by Nanjing Jiancheng Institute of Bioengineering Nanjing-China, following the instructions provided by the manufacturer.

### 2.4. Serum Indices

After 12 h of fasting on day 42, two birds were randomly selected from each replicate (16 birds per group). Blood samples were collected from each selected bird using vacutainers with clot activators. These tubes were then placed in a water bath (37 °C) for 10 min followed by centrifugation at 3000 rpm for 10 min for serum separation. These serum samples were stored at −20 °C for further analysis regarding interleukin-1 (IL-1), interleukin-2 (IL-2), interleukin-6 (IL-6), Immunoglobulin-G (IgG), and Immunoglobulin-M (IgM). These indices were determined using ELISA kits from Nanjing Jiancheng Bioengineering Institute (Nanjing, China). Collected tissue samples were analyzed for superoxide dismutase activity (SOD, cat. No. A001-2), catalase (CAT, cat. No. A007-1-1), Malondialdehyde (MDA, cat. No. A003-1-2), Glutathione (GSH, cat. No. A006-2-1), Glutathione peroxidase activity (GSH-Px, cat. No. A005-1-1) following method described by Sun et al. [19], using the commercial kits according to instructions manufacturer (Nanjing Jiancheng Bioengineering Institute, Nanjing, China).

### 2.5. Determination of Nutrient Digestibility

The gross energy and nutrient metabolic rates were determined through the total fecal collection method at 38 to 41 days. Excreta were collected for three continuous days using a scraping board while feathers, feed, and other visible impurities were picked up manually. The excreta were processed through nitrogen fixation using 10 mL of 10% hydrochloric acid/100 g of excreta. Then, they were dried at 65 °C for moisture content, which was ground, followed by passing through a 40--mesh sieve (0.45 mm), proper mixing, and stored in a sealed container until the analysis of GE, CP, CF and OM. The gross energy and chemical composition were determined according to Zhang et al. [20].

### 2.6. Relative Gene mRNA Expression

The kits for total RNA extraction (MolPure^®^ Cell/Tissue Total RNA cat. No. 19221ES50), reverse transcription (Hifair^®^ III 1st Strand cDNA Synthesis SuperMix for qPCR cat. No. 11141ES60), and real-time PCR analysis (Hieff^®^ qPCR SYBR^®^ Green Master Mix (No Rox) cat. No. 11201ES08) were purchased from Yeasen Biotechnology (Shanghai, China) Co., Ltd. The total RNA was extracted from ileum mucosal tissue according to the method provided by the manufacturer. RNA integrity was verified by determining RNA concentration and by 1% agar gel electrophoresis. Extracted 1 RNA was diluted with sterile, enzyme-free water to maintain a consistent RNA concentration for each sample. Afterward, the total RNA from each sample was reverse-transcribed into cDNA products using a reverse transcription system. The melt curve stage was programmed using the default settings of Applied Biosystems: 7500. The primer sequences used in this study are listed in Table 2. The β-actin gene was used as the internal reference gene. All samples were three replicates, and the results were analyzed using Δ^Ct^ values, and the results were calculated by the 2^−ΔΔCt^ method and expressed as the mean value [21].

### 2.7. Statistical Analysis

Data regarding all the parameters were analyzed by the General Linear Model (GLM) and one-way ANOVA using SPSS 26.0 (SPSS, Inc., Chicago, IL, USA) software, and mean comparison was carried out through LSD with significant threshold at *p* < 0.05. Data visualization in figures was achieved through Graph-Pad Prism 8 (Graph Pad Software Inc., San Diego, CA, USA) software.

## 3. Results

### 3.1. Growth Performance

**Figure 1:**
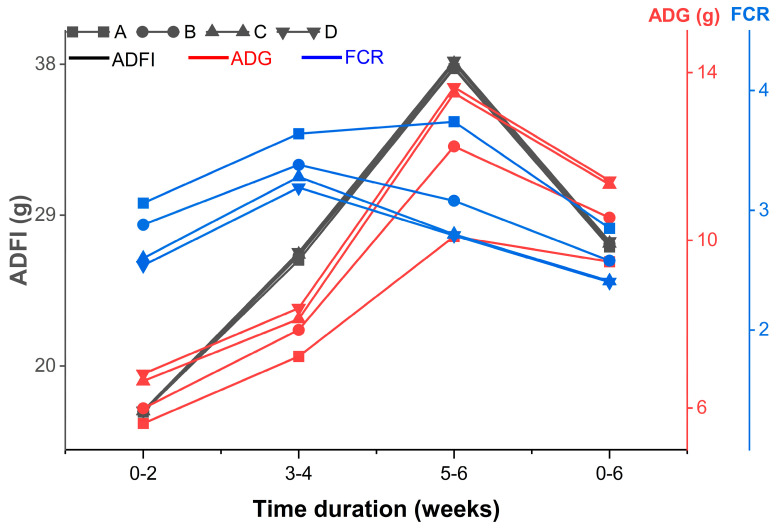
Trend of growth parameters on a fortnightly basis; A group receiving 0% Aln–Gln and also as the control group; B group receiving 0.1% Aln–Gln; C group receiving 0.2% Aln–Gln; D group receiving 0.3% Aln–Gln.

The results of the growing hens that were fed with Aln-Gln diets are summarized in Table 3. The BW and ADG were higher in growing hens that fed with Aln-Gln diets as compared to those fed control diet (*p* < 0.05) (See Figure 1).

Initially, groups C and D showed a better FCR than groups A and B, and by the end of the 6th week, the test group showed a better FCR than the control group. There was no significant difference observed between the control group and test groups regarding the ADFI during any of the experimental time intervals (*p* > 0.05).

### 3.2. Nutrient Digestibility

The nutrient digestibility data for DM, OM, CP, CF and GE on day 42 are shown in Table 4. It is important to note that the dietary treatments showed no significant effect on the digestibility of DM, OM and CF (*p* > 0.05). However, the addition of Aln-Gln positively affected GE digestibility (*p* < 0.05) and improved CP digestibility (*p* < 0.05). Notably, there was no significant difference observed between the different levels of Aln-Gln and nutrient digestibility throughout the experiment.

### 3.3. Serum Immunoglobulin and Interleukin Content

**Figure 2:**
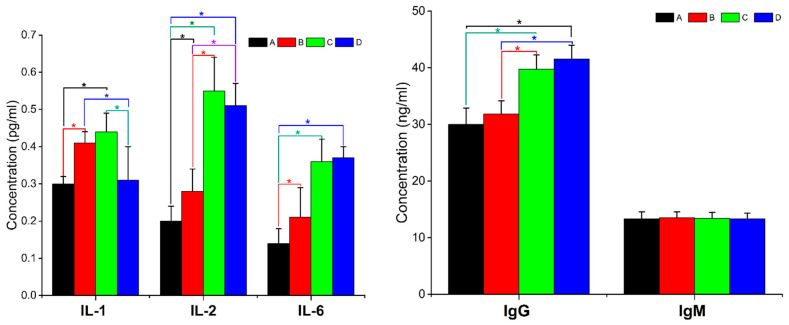
Serum immunological and interleukin content of growing hens receiving. Asterisk showed the significance of the groups at *p* < 0.05. Aln-Gln in diet; A group receiving 0% Aln-Gln and also as the control group; B group receiving 0.1% Aln-Gln; C group receiving 0.2% Aln-Gln; D group receiving 0.3% Aln-Gln.

The effect of various levels of dietary Aln-Gln supplementation on serum IgG and IgM concentrations in 42-day-old growing hens was illustrated in Figure 2. The IgG concentration in groups C and D was higher than control and group B (*p* < 0.05), while the IgM levels in groups B, C and D were similar to the control group (*p* > 0.05).

The varied effects of dietary Aln-Gln levels on serum interleukin concentration in 42-day-old growing hens was also expressed in Figure 2. The IL-1 levels in groups B and C were higher than the control and group D (*p* < 0.05). The IL-2 content in groups C and D was greater compared to the control group (*p* < 0.05), whereas, all the tested groups showed higher IL-6 as compared to the control. (*p* < 0.05).

### 3.4. Serum Antioxidant Capacity

The effect of dietary Aln-Gln supplementation on serum antioxidants like CAT, MDA, GSH-PX, GSH and SOD in 42-day-old growing hens was presented in Table 5. There was no significant difference between the tested groups and the control group (*p* > 0.05). GSH-Px showed a tendency toward significance among the groups.

### 3.5. Intestinal Digestive Enzyme Activity

Aln-Gln supplementation had no significant effect (*p* > 0.05) on the activity of amylase and lipase as compared to the control group. However, it significantly improved (*p* < 0.05) the trypsin and chymotrypsin activities in tested groups as compared to control groups (Table 6).

### 3.6. Relative Gene mRNA Expression

**Figure 3:**
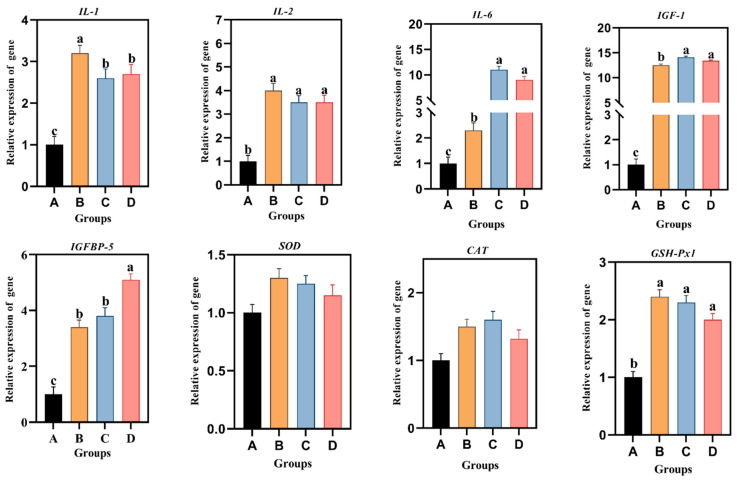
Relative Gene Expression results by qRT-PCR, presented as mean ± SEM. A group receiving 0% Aln-Gln and also as the control group; B group receiving 0.1% Aln-Gln; C group receiving 0.2% Aln-Gln; D group receiving 0.3% Aln-Gln. Means on each bar with no common letter differ significantly at *p* < 0.05.

The effects of different levels of Aln-Gln on the relative expression of genes related to growth, immunity and antioxidant status of 6-week-old *Hy-line* brown hens were expressed in Figure 3. *Hy-line* brown hens supplemented with 0.2% and 0.3% Aln-Gln of diet had a higher mRNA expression of IL-2 and IL-6 compared with groups B and A. Whereas, the addition of 0.1% Aln-Gln in the birds’ diet increased mRNA expression of IL-1 as compared to other dietary treatments (*p* < 0.05). Notably, birds that were given diets containing Aln-Gln showed a significant increase in the relative expression of IGF-1 and IGFBP-5, which are responsible for growth factors, compared to the control group (*p* < 0.05). The results showed that dietary groups showed a higher expression of GSH-Px1 as compared to the control group (*p* < 0.05). However, no significant difference was found in the relative expression of SOD and CAT-related genes among the tested groups (*p* > 0.05).

## 4. Discussion

The synthetic dipeptides of Aln-Gln are used as Gln replacement to provide an energy source in the gastrointestinal tract due to their solubility and stability. Glutamine is a vital amino acid that plays a key role in regulating protein synthesis and muscle growth [1]. This experiment studied the effects of dietary Aln-Gln dipeptide supplementation on growth performance, nutrient digestibility, digestive enzyme activity, immunity, and antioxidant status in growing laying hens. To the best of our knowledge, the effects of Aln-Gln on the performance of poultry birds were obscure. Only a few studies were carried out on Gln supplementation [2,3,4,5,6,7]. The current experiment has shown the positive effects of Gln supplementation on body weight and nutritional parameters. These results are supported by Bartell and Batal [8], who observed that the consumption of 1.0% Gln in broilers resulted in improved growth performance and an increase in IGF-1 and IGFBP-5 mRNA expression. Some previous studies have provided evidence that supplementing Gln has a positive effect on the growth performance of poultry birds and also an expression of growth-related genes [9]. In the current study, there was a significant improvement in the growth performance of growing hens, with no effect on FCR and ADFI. Improvements in body weight gain and feed efficiency have been noted in other animals, our study in line with [2,10,11,12,13,14]. The IGF-1 gene, produced in the liver, plays a crucial role in the growth of chickens. In chicks, IGF-1 found in the thigh muscles may contribute to protein synthesis through the Akt/S6 pathway [15]. All these mentioned mechanisms are primarily a result of enhanced digestive system structure and function, increased intestinal enzyme activity, improved immunity, and enhanced feed intake [16]. Glutamine being a primary metabolic fuel for enterocytes plays an important role in intestinal structural maintenance, metabolism, and physiology resulting in large absorptive surface area [11].

In the digestive system, intestinal enzyme activity is vital for the absorption and digestion of nutrients [8]. Previously, a number of studies [17,18,19] showed that disease and stressful conditions in poultry can reduce enzymatic activities. Enzymatic activity changes during animal growth may be due to digestive tract development. Our current experiment found that adding Gln in the form of Aln-Gln dipeptide to the diet increased trypsin and chymotrypsin activity in tested groups compared to the control groups. This aligns with previous studies that showed Gln addition to the poultry feed increased enzymatic activity under stressful conditions [20]. These effects were likely due to Glutamine’s interaction with food components, supporting nutrient metabolism and absorption [21]. Additionally, the increased enzymatic activities may be attributed to improved secretion in the pancreas, intestinal health, and increased expression of heat shock protein 70 due to dietary Glutamine supplementation, which could subsequently enhance broiler growth performance [20].

A limited number of studies are available on the effects of Gln on the nutrient digestibility of growing laying hens [22,23,24]. We found that feeding 0.2% and 0.3% Gln in the hen’s diet significantly increases the digestion of CP and GE in the growing phase. As we described earlier, Gln improved the digestive enzymatic activities and gut morphology, being a key component of nutrient digestion, in line with the findings [24].

Antibodies, also known as immunoglobulins, are important glycoprotein molecules produced by B lymphocytes [25]. Our findings depicted that hens supplemented with Aln-Gln showed increased levels of IgM in serum, while IgG levels remained unaffected. During the growing phase, rapid changes in the body’s immunoglobulin levels may lead to acute stress reactions [26]. Gln’s ability to protect immunity may take part in the reduction of peripheral immune cells. Advanced research is needed to deeply understand the potential uses of Gln in the feed industry [27].

The compound MDA serves as a marker of lipid peroxidation and represents a stable end product of lipid oxidation in broiler muscle. According to Wu et al. [25], MDA is indicative of the oxidative stress damage resulting from stress. A study observed that dietary Gln did not significantly affect MDA levels in serum, suggesting a clear reduction in lipid peroxidation. The SOD, GSH-Px, CAT and GSH are enzymatic antioxidants present in the cells of animal’s body. The antioxidant content and activity indirectly indicate the body’s capacity to eliminate free radicals [21]. In a study, it was found that the levels of GSH-Px, CAT, GSH and SOD in the serum of growing hens remained unchanged after the addition of Gln and there was no significant difference in mRNA expression of related genes [24]. Furthermore, it was observed that the supplementation of Gln led to a slight increase in the gene expression levels of GSH-Px [28,29]. Similarly, Hu et al. reported that adding Gln in the diet improved antioxidant capacity and reduced lipid peroxidation by activating antioxidant-related gene expression in broilers exposed to cyclic heat stress [30].

The production of pro-inflammatory cytokines is essential for innate host defense system activation and subsequently controlling the adaptive immune response [31]. The cytokine-related genes, including IL-6, TNF-α and IL-1β, have a substantial effect on the above-mentioned mechanisms. The IL-6 and IL-1 genes, which are produced by macrophages, play a significant role in various inflammatory reactions [32,33]. Our current findings demonstrated that the addition of Aln-Gln resulted in elevated mRNA levels of IL-2, IL-6 and IL-1 genes in blood, suggesting the initiation of an acute inflammatory response. It was shown that stress led to increased mRNA expression of TNF-α, IL-6 and IL-1 genes in the broilers. In the growth period of *Hy-line* brown hens, a rise in mRNA expressions of IL-6, IL-2 and IL-1 was reported [34,35,36]. Another study indicated that supplementing with Glutamine raises the levels of TNF-α, IL-6 and IL-1β in the plasma of broiler chickens exposed to LPS [37,38]. Furthermore, it has been demonstrated that a lack of Glutamate reduces the production of pro-inflammatory cytokines, whereas, the addition of Glutamate limits the inflammatory response in laboratory experiments. Overall, these findings suggest that supplementing with Gln may contribute to a reduction in inflammatory reactions caused by various stressed conditions [39]. Additionally, the positive effects of Gln supplementation, such as enhanced muscle protein synthesis, causes changes in the concentrations of corticosterone, T3, T4, and insulin, and activation of metabolic enzymes in the serum. Furthermore, there is modulation of the hypothalamic-pituitary-adrenal axis, inhibition of the central nervous system neurotransmitters, as well as a reduction in the number of intra-epithelial leukocytes in the intestinal mucosa of poultry birds [40,41,42,43].

Our study has shown that Gln can improve early life growth performance under varying conditions. It is thought that this improvement is due to Glutamine’s positive effects on the growth and development of digestive organs, nutrient digestion, and apparent nitrogen retention [44,45,46]. Additionally, Gln may be involved in the betterment of intestinal mucosa and might enhance protein synthesis efficiency or other physiological requirements in poultry diets.

## 5. Conclusions

In conclusion, we have demonstrated that a 0.2% level of Aln-Gln supplementation in *Hy-Line* brown hens during the growing period improved growth, nutrient digestibility, immunity, and digestive enzymatic activity. These findings suggest that Aln-Gln is a promising dietary additive in the enhancement of poultry performance.

## Figures and Tables

**Table 1:** Composition and nutrient levels of diets on dry matter basis (0–6 weeks).

Ingredients (%)	A	Addition of Aln-Gln Powder		
		B	C	D
Corn	66.100	66.000	66.000	65.900
Soybean	29.000	29.000	28.900	28.990
Wheat Bran	0.200	0.200	0.200	0.200
Alanyl Glutamine Powder	-	0.100	0.200	0.300
Met	0.200	0.200	0.200	0.200
Lys	0.200	0.200	0.200	0.200
Salt	0.300	0.300	0.300	0.300
Limestone	1.300	1.300	1.300	1.300
CaHPO_3_	1.700	1.700	1.700	1.700
Premix	1	1	1	1
Total	100	100	100	100
ME (MJ/Kg)	11.98	11.98	11.98	11.98
CP %	18.4	18.4	18.4	18.4
Lys Dig. %	1.126	1.126	1.126	1.126
Met Dig. %	0.47	0.47	0.47	0.47
Thr Dig. %	0.68	0.68	0.68	0.68
Trp Dig. %	0.20	0.20	0.20	0.20
Arg Dig. %	1.22	1.22	1.22	1.22
Ile Dig. %	0.74	0.74	0.74	0.74
Val Dig. %	0.84	0.84	0.84	0.84
CF %	2.78	2.78	2.78	2.78
Ca %	1.078	1.078	1.078	1.078
P Av. %	0.45	0.45	0.45	0.45
Na %	0.023	0.023	0.023	0.023
Cl %	0.041	0.041	0.041	0.041

Per kilogram of diet, the following are provided: 11,000 IU of vitamin A, 3000 IU of vitamin D3, 20 IU of vitamin E, 3 mg of vitamin K3 (as menadione), 0.02 mg of vitamin B12, 6.5 mg of riboflavin, 10 mg of calcium pantothenate, 40.1 mg of niacin, 0.2 mg of biotin, 2.2 mg of thiamine, 4.5 mg of pyridoxine, 1000 mg of choline, 125 mg of ethoxyquin (antioxidant). Additionally, per kilogram of diet, the following are provided: 66 mg of Mn (as manganese oxide), 70 mg of Zn (as zinc oxide), 80 mg of Fe (as ferrous sulfate), 10 mg of Cu (as copper sulfate), 0.3 mg of Na (as sodium selenite), 0.4 mg of I (as calcium iodate), 0.67 mg of iodized salt.

**Table 2:** Primer sequences for the genes utilized in the qRT-PCR.

Name	Details	Gene ID	Product Length	Primer
IL1	interleukin 1	100861585	136	F: GGATGGTTCCTCTGCACCTC R: GGGAGCAGAGCGCCTTTATT
IL2	interleukin 2	395294	109	F: CCGACCGGCTACAATAAGCA R: CGCTGTCATTGGTACATGGC
IL6	interleukin 6 receptor	693252	92	F: CAGCCACGACAAAGATGTGC R: TGAACCTGCGCTTCATCCAT
IGF-1	insulin like growth factor 1	395889	83	F: GCAGTAGACGCTTACACCACAAGG R: ACAGTACATCTCCAGCCTCCTCAG
IGFBP-5	insulin-like growth factor binding protein5	424220	128	F: GCGACCGAAAGGGATTCTACAAGAG R: CAGGTCTCCGCTCAGGTAGTCAG
SOD	superoxide dismutase 1	395938	73	F: CTTACCGGACCACACTGCAT R: CCCCTCTACCCAGGTCATCA
CAT	catalase	423600	142	F: TGCATCATTGGCCGTACCAT R: ACAACGGTTAGCACTTGGCT
GSH-Px1	Glntathione peroxidase	130713843	124	F: TCACCATGTTCGAGAAGTGC R: ATGTACTGCGGGTTGGTCAT
β-actin	actin, beta	396526	144	F: CTACACACGGACACTTCAAG R: ACAAACATGGGGGCATCAG

**Table 3:** Growth performance of growing hens from 0–6 weeks.

Period	Groups				SEM	*p*-Value
	A	B	C	D		
**Intial BW** (g)						
1 day	38.72	38.80	39.01	38.74	2.331	0.821
**BW** (g)						
2 wk	117.54 ^c^	122.66 ^b^	131.97 ^a^	134.08 ^a^	1.240	0.021
4 wk	257.50 ^c^	271.32 ^b^	286.62 ^a^	288.31 ^a^	2.258	0.012
6 wk	437.54 ^c^	481.41 ^b^	514.58 ^a^	518.47 ^a^	5.831	0.025
**ADFI** (g/bird/d)						
0–2 wk	17.26	17.28	17.31	17.33	0.041	0.980
3–4 wk	26.31	26.57	26.66	26.78	0.046	0.593
5–6 wk	37.74	37.81	38.04	38.19	0.641	0.312
0–6 wk	27.11	27.27	27.31	27.43	1.310	0.074
**ADG** (g/bird/d)						
0–2 wk	5.63 ^c^	5.99 ^b^	6.64 ^a^	6.81 ^a^	0.875	0.010
3–4 wk	7.23 ^b^	7.86 ^b^	8.12 ^a^	8.38 ^a^	0.079	0.021
5–6 wk	10.09 ^c^	12.24 ^b^	13.51 ^a^	13.65 ^a^	0.258	0.005
0–6 wk	9.49 ^c^	10.54 ^b^	11.33 ^a^	11.42 ^a^	0.138	0.017
**FCR**						
0–2 wk	3.06 ^a^	2.88 ^a^	2.60 ^b^	2.54 ^b^	0.039	0.019
3–4 wk	3.64 ^a^	3.38 ^b^	3.28 ^c^	3.19 ^c^	0.331	0.032
5–6 wk	3.74 ^a^	3.08 ^b^	2.80 ^c^	2.79 ^c^	0.678	0.013
0–6 wk	2.85 ^a^	2.58 ^b^	2.41 ^c^	2.40 ^c^	1.173	0.041

a, b, c, are shown in the same row with different letters differ significantly at *p* < 0.05. A group receiving 0% Aln–Gln and also as the control group; B group receiving 0.1% Aln–Gln; C group receiving 0.2% Aln–Gln; D group receiving 0.3% Aln–Gln. **Abbreviations**: BW, body weight; ADG, average daily gain; ADFI, average daily feed intake; FCR, feed conversion ratio.

**Table 4:** Nutrient digestibility of growing laying hens fed Aln-Gln-containing diets (%).

Parameters	Groups				SEM	*p*-Value
	A	B	C	D		
Dry matter	72.35	71.51	73.16	72.87	4.24	1.060
Organic matter	70.12	71.33	71.11	69.90	1.39	0.733
Crude Protein	56.10 ^b^	63.97 ^a^	62.44 ^a^	60.01 ^ab^	0.92	0.021
Crude Fat	76.44	75.87	78.21	77.91	6.43	0.982
Gross Energy	65.11 ^b^	71.22 ^a^	69.95 ^a^	71.90 ^a^	5.32	0.040

a, b, showed in the same row with different letters differ significantly at *p* < 0.05. A group receiving 0% Aln–Gln and also as the control group; B group having 0.1% Aln–Gln; C group receiving 0.2% Aln–Gln; D group receiving 0.3% Aln–Gln.

**Table 5:** Serum antioxidant capacity of growing hens.

Parameters	Groups				SEM	*p*-Value
	A	B	C	D		
CAT (U/mL)	1.20	1.25	1.21	1.27	0.01	0.268
MDA (nmol/mL)	3.11	3.23	3.19	3.17	0.39	0.103
GSH-Px (U/mL)	210.40	212.01	213.48	209.91	0.21	0.090
GSH (U/mL)	6.78	6.68	6.59	6.67	0.24	0.981
SOD (U/mL)	271.33	272.42	269.65	270.20	9.80	0.271

A group receiving 0% Aln-Gln and also as the control group; B group receiving 0.1% Aln-Gln; C group receiving 0.2% Aln-Gln; D group receiving 0.3% Aln-Gln. **Abbreviations:** CAT, catalase; GSH, Glntathione; GSH-Px, Glntathione peroxidase; MDA, malondialdehyde; SOD, superoxide dismutase.

**Table 6:** Digestive enzymes activities of growing hens.

Items	Groups				SEM	*p*-Value
	A	B	C	D		
Trypsin activity (U/mg prot)	615.04 ^c^	633.89 ^b^	649.05 ^a^	644.54 ^ab^	17.40	0.020
Chymotrypsin activity (U/mg prot)	2.27 ^b^	3.51 ^a^	3.39 ^a^	3.42 ^a^	0.95	0.031
Lipase Actvity (U/mg prot)	4.78	4.81	4.58	4.27	1.23	0.446
Amylase Activity (U/mg prot)	0.21	0.19	0.23	0.18	0.01	1.347

a, b, c, showed in the same row with different letters differ significantly at *p* < 0.05. A group receiving 0% Aln-Gln and also as the control group; B group receiving 0.1% Aln-Gln; C group receiving 0.2% Aln-Gln; D group receiving 0.3% Aln-Gln.

## Data Availability

The datasets supporting the conclusions of this article are included within the article.
